# Protease-Activated Receptor-1 Supports Locomotor Recovery by Biased Agonist Activated Protein C after Contusive Spinal Cord Injury

**DOI:** 10.1371/journal.pone.0170512

**Published:** 2017-01-25

**Authors:** William D. Whetstone, Breset Walker, Alpa Trivedi, Sangmi Lee, Linda J. Noble-Haeusslein, Jung-Yu C. Hsu

**Affiliations:** 1 Department of Emergency Medicine, University of California, San Francisco, California, United States of America; 2 Department of Neurological Surgery, University of California, San Francisco, California, United States of America; 3 Department of Physical Therapy and Rehabilitation Science, University of California, San Francisco, California, United States of America; 4 Department of Cell Biology and Anatomy, College of Medicine, National Cheng Kung University, Tainan City, Taiwan; University of Toronto, CANADA

## Abstract

Thrombin-induced secondary injury is mediated through its receptor, protease activated receptor-1 (PAR-1), by "biased agonism." Activated protein C (APC) acts through the same PAR-1 receptor but functions as an anti-coagulant and anti-inflammatory protein, which counteracts many of the effects of thrombin. Although the working mechanism of PAR-1 is becoming clear, the functional role of PAR-1 and its correlation with APC in the injured spinal cord remains to be elucidated. Here we investigated if PAR-1 and APC are determinants of long-term functional recovery after a spinal cord contusive injury using PAR-1 null and wild-type mice. We found that neutrophil infiltration and disruption of the blood-spinal cord barrier were significantly reduced in spinal cord injured PAR-1 null mice relative to the wild-type group. Both locomotor recovery and ability to descend an inclined grid were significantly improved in the PAR-1 null group 42 days after injury and this improvement was associated with greater long-term sparing of white matter and a reduction in glial scarring. Wild-type mice treated with APC acutely after injury showed a similar level of improved locomotor recovery to that of PAR-1 null mice. However, improvement of APC-treated PAR-1 null mice was indistinguishable from that of vehicle-treated PAR-1 null mice, suggesting that APC acts through PAR-1. Collectively, our findings define a detrimental role of thrombin-activated PAR-1 in wound healing and further validate APC, also acting through the PAR-1 by biased agonism, as a promising therapeutic target for spinal cord injury.

## Introduction

Spinal cord injury results in direct vascular damage, followed by an inflammatory response and a cascade of events that further disrupt the blood-spinal cord barrier. One of the earliest factors produced following injury is the serine protease thrombin [1, 2], which is a 36 kDa protein comprised of two chains, A and B, that are linked by a disulfide bond [3]. Thrombin primarily contributes to coagulation by the conversion of fibrinogen to fibrin and the activation of platelets and several coagulation factors [4–6]. It is also known to mediate inflammation and endothelial permeability [7, 8].

Thrombin acts through at least four different receptor subtypes, called protease activated receptors (PARs), that are designated PAR-1, -2, -3, and -4 [9–12]. In the CNS, PAR-1 is expressed on motoneurons [13, 14], astrocytes, and cerebral endothelial cells [15, 16]. When activated by thrombin, PAR-1 has multiple detrimental effects, including death of motor neurons, neurite retraction, and astrogliosis [15]. Moreover, thrombin-activated PAR-1 in the endothelium not only induces pro-inflammatory signaling but also alters the cell shape, increases vascular permeability, and modulates leukocyte trafficking [17, 18]. In fact, PAR-1 induces several adhesion molecules such as P-selectin in endothelial cells, which contribute to the recruitment of inflammatory cells and subsequent secondary damage to vascular tissue [19].

Thrombin is modulated by several factors including activated protein C (APC), which is created when thrombomodulin binds to thrombin and protein C. Unlike thrombin, APC functions as an anti-coagulant but acts through the same PAR-1 receptor. [20–24]. However, subsequent signaling of APC results in opposite effects to that of thrombin. Convergent evidence indicates that APC reduces coagulation and inflammation, stabilizes the vascular barrier, enhances neuroprotection and neurological recovery in experimental models and clinical trials such as stroke, traumatic brain injury, and compressive spinal cord injury [25–29].

The diverse effects of thrombin and APC may reflect “biased agonism” [12] whereby activation of PAR-1 by thrombin or APC involves distinct intracellular signaling cascades [30, 31]. In endothelium, for example, thrombin-PAR-1 activates GTPase RhoA, but not the related protein Rac1 [32], a cascade known to disrupt endothelial barrier integrity [33]. In contrast, APC-PAR-1 activates Rac1, but not RhoA [32] thus enhancing barrier integrity [12, 34].

Here we consider the concept of biased agonism and test the hypothesis that thrombin-PAR-1 activation impedes wound healing and recovery after spinal cord injury, while the same receptor activated by APC improves functional recovery. We compared the leukocyte infiltration, vascular barrier disruption, glial scar formation, axonal sparing, and locomotor recovery between PAR-1 null and wild-type mice. Our results not only implicate PAR-1 in early secondary injury, but also confirm the potential of APC as an effective treatment for spinal cord injury, suggesting that biased agonism can be favorably exploited to improve outcome after spinal cord injury.

## Materials and Methods

All procedures involving animals were approved by the UCSF Institutional Animal Care and Use Committee and in accordance with the National Institutes of Health Guide for the Care and Use of Laboratory Animals. PAR-1 null mice were a generous gift from Dr. Sean Coughlin (Cardiovascular Research Institute, University of California, San Francisco) and bred on a C57Bl/6 background. Adult PAR-1 null mice have been extensively characterized and demonstrate normal physiology [35, 36]. All efforts were made to minimize animal suffering.

### Surgery and animal care

Adult male mice (n = 100) weighing between 30 and 35g were anesthetized with 250 mg/kg body weight of 2.5% tribromoethanol. Body temperature was maintained during surgery by means of a warming blanket. Animals were subjected to a moderate spinal cord contusive injury at the mid-thoracic level as we have previously described [37]. Briefly, a laminectomy was performed at the T8 vertebra to expose the spinal cord. The animal was then positioned in a modified contusion device with forceps attached to the spinous processes of T7 and T9. An impactor tip was carefully positioned on the surface of the exposed cord and a 3 g weight was dropped 5 cm onto the impactor tip. Animals, subjected to laminectomy only, served as sham controls. Postoperative care included subcutaneous administration of antibiotics sulfamethoxizole-trimethoprim (0.9 mg/ml) and manual expression of the bladder twice per day.

### Histology

Mice were re-anesthetized and transcardially perfused with 4% paraformaldehyde in phosphate buffered saline (PBS) at either 1 or 42 days after injury. The vertebral column was removed and post-fixed in 4% paraformaldehyde for 3 hours at 4°C. Then the spinal cord was extracted and cryoprotected in 20% sucrose in PBS for 3 days at 4°C. A 15-mm length of cord, centered over the impact site was divided into 3 segments of equal length. Each segment was embedded in tissue freezing medium and cut transversely at 20-μm in thickness on a cryostat.

Sections were prepared for immunofluorescence or stained with luxol fast blue. Neutrophils were immunolocalized using a 1:1600 dilution of rat anti-mouse neutrophil antibody (GR1, Caltag, Burlingame, CA) and visualized by a CY3 conjugated goat anti-rat secondary antibody. Astrocytes were localized with mouse anti-glial fibrillary acidic protein (GFAP 1:400, clone G-A-5, G3893; Sigma, St. Louis, MO) and CY3-conjugated mouse anti-GFAP (1:400, clone G-A-5, C9205; Sigma, St. Louis, MO). Endothelium of blood vessels were localized using rat anti-platelet/endothelial cell adhesion molecule-1 (PECAM-1, a.k.a. CD31, 1:400, clone MEC13.3, 550274; BD Biosciences, San Jose, CA). PAR-1 was immunolocalized with goat anti-human PAR-1, recognizing the amino and carboxy termini (1:100, Santa Cruz Biotechnology, Santa Cruz, CA), followed by a 1:200 dilution of biotinylated rabbit anti-goat secondary antibody (Jackson Immunoresearch, Westgrove, PA). Binding was visualized with Fluorescein Avidin D or Texas red-avidin D (1:100 in PBS; Vector Laboratories, Burlingame, CA).

### Quantification of neutrophils

Infiltration of neutrophils was assessed at 24 hours after spinal cord injury in each of the genotypes (n = 28). The epicenter of injury was defined by dark field microscopy. Five cross sections of the spinal cord were prepared from each of the 3 regions including the epicenter, 3 mm rostral to the epicenter, and 3 mm caudal to the epicenter. After neutrophil immunostaining, each of these sections was photographed with a 4× objective using a Leica microscope equipped with a CCD camera (SPOT software, model 1.3.0; Diagnostic Instruments, Inc. Sterling Heights, MI) and imported into Stereo Investigator (MicroBrightField, Williston, VT). A contour was drawn closely around the entire section. An observer, blinded to the experimental condition, counted all neutrophils in the section. For accuracy of counting, the images were enlarged to 40× to better identify clustered cells.

### Luciferase assay

At 24 hours after surgery, mice (n = 8/group) were re-anesthetized and the integrity of the vascular barrier was quantified as we have previously described [37]. Each mouse was injected with luciferase (Sigma, St. Louis, MO) through the jugular vein (100 μl/30 g body weight). At 25 minutes after the injection, 20 μl of venous blood was obtained and diluted in 80 μl of PBS. At 30 minutes after the injection, each animal was intracardially perfused with PBS and the cord was extracted. Both the cord and blood sample were quickly frozen on dry ice.

A 9 mm length of cord, centered over the lesion epicenter, was divided into 3 segments of equal length and weighed. Each segment was then homogenized in cell lysis buffer at a 1:50 dilution. The homogenate was subjected to centrifugation (8 minutes at 12,000 rpm) and 15 μl of supernatant was collected and incubated in 1,400 μl of cell lysis buffer at room temperature for one hour. Then 15 μl of sample was added to 100 μl of substrate from the Luciferase Assay System (Promega, Madison, WI) and mixed by pipette. The luminosity of the reaction was measured in a TD 20/20 luminometer (Turner Designs, Sunnyvale, CA). Values were expressed as percent of the luminosity, as determined in the blood sample.

### Functional assessment

Several tests were used to assess recovery of function in spinal cord injured animals (n = 10/group). These included quantitative assessment of locomotion in an open field and ability to traverse a grid, and qualitative assessment of hindlimb paw position. A trained observer, who was blinded to the animal identity, assessed motor recovery in an open field using the Basso Mouse Scale for locomotion [38] at 1 and 3 days after injury and weekly thereafter for six weeks. Each mouse was videotaped during testing.

For three sequential days starting 35 days after injury, each mouse was tested on a grid of 20 parallel bars with 20-mm spacing and a 30-degree plane of inclination [39]. The mouse was videotaped and evaluated in slow motion by 2 blinded observers who counted each time the mouse intentionally gripped the bar while descending.

Analysis of hindlimb placement was studied in those animals that scored at 4 or higher on the open field testing paradigm. At 42 days after injury, the hind feet were painted with non-toxic paint. The mouse then traversed a Lucite tunnel (60 cm in length) lined with paper. Impressions of pawprints were obtained for each experimental group.

### Assessment of white matter sparing

Sparing of white matter, a correlate of motor recovery [40], was quantified in animals euthanized at 42 days post injury (n = 7/group). Every fifth section from the lesion epicenter was stained with 0.1% luxol fast blue in 0.5% acetic acid at 60°C for 3 hours, followed by 0.05% lithium carbonate for 3 minutes to visualize the white matter. The sections were then photographed at 100× magnification and their contours were delineated around the residual white matter as well as the circumference of the cross section to measure the area using the Neurolucida imaging system (MicroBrightField, Williston, VT). The section with the least residual white matter was defined as the epicenter of the injury.

### Assessment of the astrocytic scar

Astrocytic scarring was evaluated in sections immunolabeled for GFAP at 42 days post-injury (n = 6/group). The severity of the glial scarring was analyzed by a semi-quantitative method first described by Hsu et al. [40]. Briefly, the transverse section of the injured spinal cord was subdivided into 12 sectors by superimposing a grid over the entire digitized image of the section using Photoshop CS (Adobe Systems Inc., San Jose, CA). In each sector, the complexity of the astrocytic scar was evaluated based on the distribution of astrocytes, arborization and organization of astrocytic processes, astrocytic hypertrophy, and the intensity of GFAP immunoreactivity. Then a score ranging from 0 to 3 was given for each sector. A score of 0 indicates no evidence of glial scar formation, whereas a score of 1 to 3 represents increasing complexity of the glial scarring. Three serial sections at intervals of 480 μm were sampled from the lesion epicenter in each animal. In each sample section, the score obtained from all 12 sectors represented the severity (complexity and/or extent) of the glial scarring and the total score of 3 sample sections was tallied for subsequent comparison between mouse groups.

### Administration of APC

Each of the wild-type and PAR-1 null groups was further divided into two subgroups. Based on the dosage of a previous study [41], one subgroup (n = 7) was given recombinant (r) APC (2 mg/kg body weight, Eli Lilly, Indianapolis, IN) dissolved in saline as a single 0.3-ml bolus intravenously 20 minutes after injury, followed immediately by an additional 0.3-ml subcutaneous dose. The other subgroup (n = 7) received saline vehicle the same way to serve as the control group. Motor recovery for mice treated with rAPC or control vehicle was assessed using the Basso Mouse Scale as described above.

### Statistical analyses

Limited numbers of animals died during the course of experiments. Two mice died in each of the saline-treated wild-type, APC-treated wild-type, and saline-treated PAR-1 null groups 1 days after the injury. One mouse died in the saline-treated PAR-1 null group 3 days after the injury and in the APC-treated PAR-1 null group 1 day and 3 days, respectively, after the injury. Data obtained from deceased animals were excluded from statistical analyses. To reduce bias, all quantitative measurements were assessed by designated observers blinded to the animal identity, genotypes, and treatments. Then a second researcher knowing the keys that identify animal subjects collected the data for further statistical analyses. Comparisons between PAR-1 null and wild-type groups were made by unpaired Student's *t* tests to analyze neutrophil infiltration, luciferase permeability, glial scarring, residual white matter, and grid walking, whereas repeated 2-way ANOVA, followed by Bonferroni's *post hoc* test, was performed for multiple comparisons between groups to evaluate locomotor recovery. The mean values ± SEM were reported. Statistical significance was defined at *P* ≤ 0.05.

## Results

### PAR-1 is expressed by neurons and endothelial cells in uninjured cord and is up-regulated in astrocytes after injury

**Fig 1:**
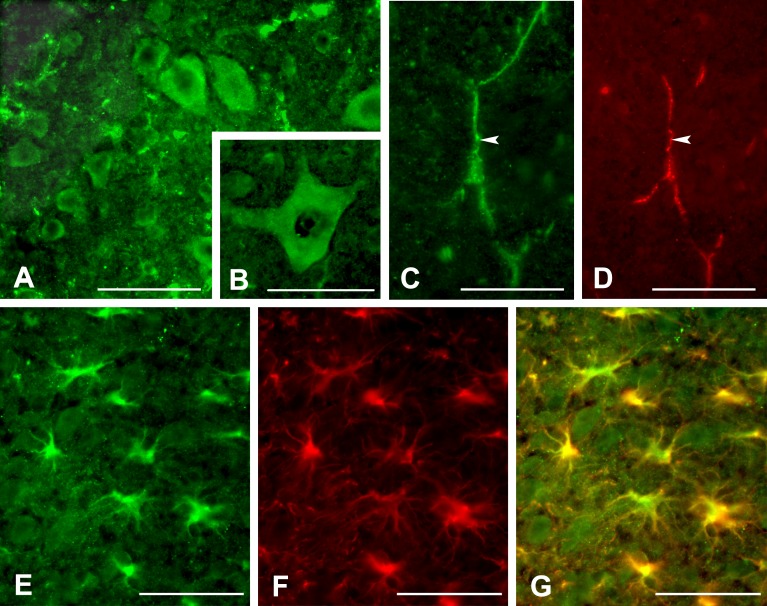
Immunolocalization of PAR-1 in the spinal cord of the wild-type mice. PAR-1 is expressed by neurons in the ventral horn in the uninjured spinal cord (A). At higher magnification, the PAR-1-positive neuron exhibits typical multipolar morphology of the spinal motor neurons (B). PAR-1 (arrow, C) also co-localizes with PECAM-1-positive capillaries (arrow, D) in the uninjured cord. After spinal cord injury, PAR-1 is expressed by reactive astrocytes 24 hours post-injury (E). These reactive astrocytes in the lesion show increased expression of GFAP and hypertrophic morphology (F) as demonstrated in the digitally merged image (G). Scale bars = 100 μm for A, C, D; 50 μm for B, E, F, G.

We first identified the cell types that express PAR-1 in the spinal cord of wild-type mice. PAR-1 was immunoexpressed in ventral horn motoneurons and co-localized with PECAM-1, a vascular marker, in the uninjured spinal cord (Fig 1A–1D). Specificity of the anti-PAR-1 antibody was confirmed using both a blocking antibody as well as omission of the primary antibody (data not shown). While PAR-1 was not localized to astrocytes in the uninjured cord, it was expressed in reactive astrocytes, featured by increased GFAP immunoreactivity and hypertrophic cell bodies, at the lesion epicenter by 24 hours post injury (Fig 1E–1G).

### PAR-1 null mice show reduced neutrophil infiltration and improved vascular integrity after spinal cord injury

**Fig 2:**
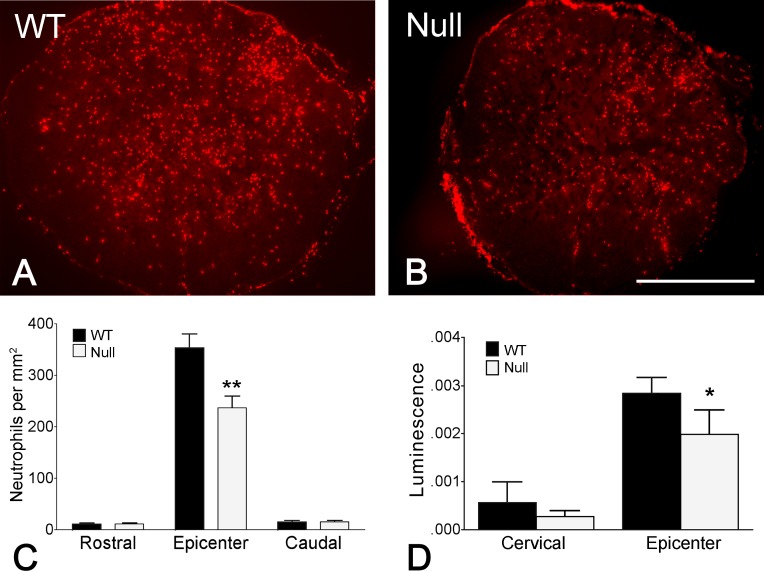
Reduced infiltration of neutrophils and disruption of vascular barrier in the injured spinal cord in PAR-1 null mice. Aggregation of immunolabled GR1-positive neutrophils is evident in the lesion epicenter in both wild-type (A) and PAR-1 null mice (B) 24 hours after injury. Quantitative analysis reveals that the number of infiltrated neutrophils within the lesion epicenter is significantly lower in the PAR-1 null mice than in the wild-type mice (C). Such a difference in number between the 2 groups of mice is not apparent in segments rostral or caudal to the epicenter. The luciferase luminescence, which inversely represents the integrity of the blood-spinal cord barrier, is significantly decreased at the lesion epicenter in PAR-1 null mice as compared to that of the wild-type mice. (n = 7/genotype, means ± SEM, unpaired Student's *t*-test, *p < 0.05, **p < 0.01).

To evaluate the role of PAR-1 in early inflammatory responses and the integrity of the vascular barrier after spinal cord injury, we examined the extent to which neutrophils infiltrated into the cord parenchyma and the permeability of blood vessels in the lesion using both PAR-1 null and wild-type mice. Neutrophils were immunolocalized in the injured cord at 24 hours post injury and were most prominent within the lesion epicenter in both groups of mice (Fig 2A and 2B). The numbers of neutrophils were quantified at the epicenter as well as segments both rostral and caudal to the epicenter. We found that the number of infiltrated neutrophils was significantly higher in the epicenter than in the rostral and caudal segments in both groups of mice (Fig 2C). Remarkably, PAR-1 null mice showed significantly fewer neutrophils within the epicenter as compared to the wild-type group (Fig 2C).

We have previously shown that spinal cord injury results in a profound disruption of the blood-spinal cord barrier to the protein luciferase [37]. To determine if PAR-1 is a determinant of this abnormal vascular permeability, we compared barrier disruption to luciferase in wild-type and PAR-1 null mice 24 hours after injury. Luminescence, indicative of blood-spinal cord barrier disruption to luciferase, was greatest within the injury epicenter, as compared to segments rostral to the epicenter in both groups of mice (Fig 2D). Moreover, PAR-1 null mice had significantly lower luminescence in the epicenter than wild-type mice (Fig 2D). Our findings suggest a detrimental role of PAR-1 in promoting early inflammation and vascular barrier breakdown after spinal cord injury.

### PAR-1 null mice show better locomotor recovery, more spared white matter, and reduced glial scarring

**Fig 3:**
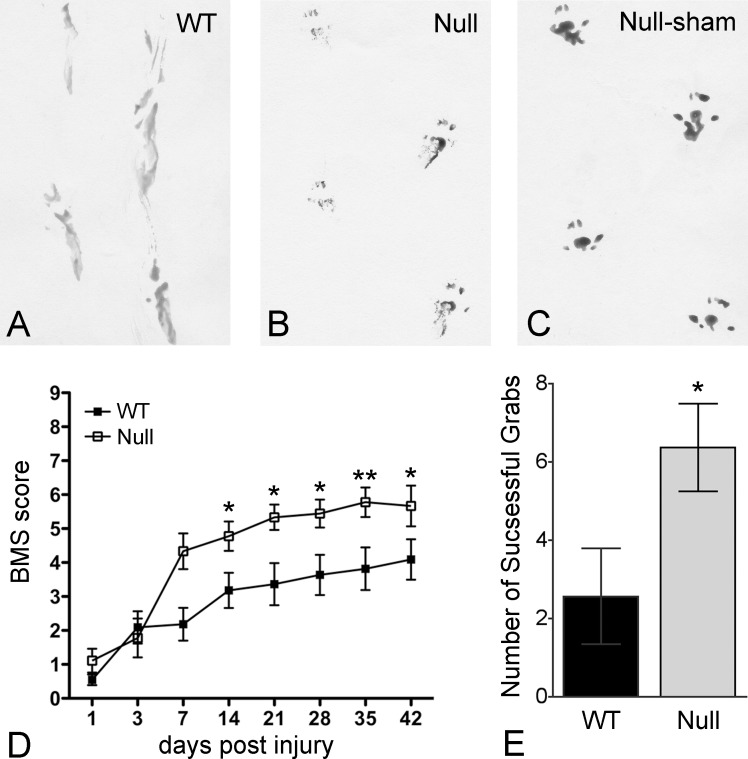
Improved motor function recovery in PAR-1 null mice after spinal cord injury. Representative pawprints show the walking patterns of the injured wild-type (A) and PAR-1 null mice (B) 42 days post injury as well as that of the uninjured PAR-1 null mice (C). After spinal cord injury, dragging of the hindlimbs with poor coordination is evident in the wild-type mice, whereas considerable improvement with weight-bearing stepping and slight external rotation of the hindpaws is consistent in the PAR-1 null mice. Injured PAR-1 null mice also show significant locomotor recovery, assessed by the Basso Mouse Scale, as compared to the wild-type mice (D). This result parallels the better performance of PAR-1 null mice on the inclined grid (E). (n = 10/genotype, means ± SEM, 2-way ANOVA for locomotor assessment, unpaired Student's *t*-test for inclined grid, *p < 0.05, **p < 0.01).

As reduction in infiltrated neutrophils and early stabilization of the vascular barrier has been associated with long-term neurological improvement, we next examined functional recovery after spinal cord injury in both PAR-1 null and wild-type mice using a series of behavioral tests including a pawprint analysis to study the stepping pattern, an open-field testing paradigm, the Basso Mouse Scale [38], to assess locomotion, and an inclined grid to evaluate accurate fine motor control in descending the grid (Fig 3). PAR-1 null mice displayed an improved gait pattern with weight-bearing stepping and coordination as compared to wild-type mice, which showed consistent dragging of hindlimbs in pawprint analysis at 42 days after injury (Fig 3A–3C). Moreover, PAR-1 null mice showed a marked improvement in locomotion beginning 14 days post injury and for the remainder of the testing period (Fig 3D). Performance on an inclined grid was likewise significantly improved in the PAR-1 null group (6.3 ± 1.1 successful grabs) as compared to wild-type group (2.5 ± 1.2 successful grabs) 35 days after injury (Fig 3E).

**Fig 4:**
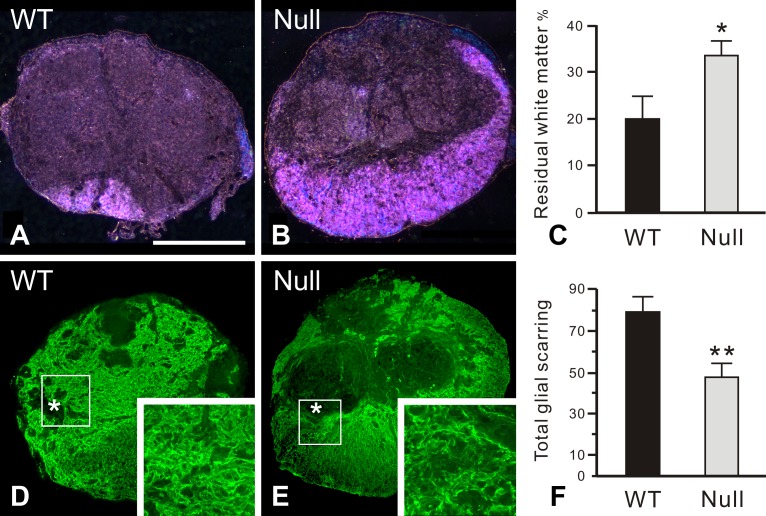
Enhanced white matter sparing and reduced glial scar formation at the lesion epicenter in PAR-1 null mice 42 days post injury. Residual white matter is visualized by luxol fast blue staining using dark-field microscopy. Wild-type mice (A) have less residual white matter, mainly located at the ventral-most part of the spinal cord cross section, than the PAR-1 null mice (B). Such a difference in the size of spared white matter is statistically significant (C). The glial scar, characterized by intense GFAP immunoreactivity, is more widespread at the lesion epicenter in the wild-type mice (D) relative to the PAR-1 null mice (E). Boxed areas enclose part of the glial limitans, an interface separating the GFAP-quiescent areas (asterisks) in the lesion epicenter from the residual cord tissue. At higher magnification, more densely entangled astrocytic processes are apparent in the wild-type mice than in the PAR-1 null mice as demonstrated in the insets. As described in Materials and Methods, the quantitative analysis demonstrates that the total score of the glial scarring, which represents the severity of glial scar formation, is significantly lower in the PAR-1 null mice than in the wild-type controls (F). Scale bar = 500 μm. (n = 7 and 5/genotype for the measurement of spared white matter and glial scarring, respectively, means ± SEM, unpaired Student's *t*-test, *p < 0.05, **p < 0.01).

At the completion of the behavioral tests, we further determined the size of the cross-sectional area of the spinal cord and the area of residual white matter stained by luxol fast blue at the lesioned epicenter. Although the cross-sectional areas of the injured cords were comparable between the 2 groups, PAR-1 null mice possessed significantly more residual white matter (34 ± 4%) than the wild-type mice (20 ± 6%) 42 days after injury (Fig 4A–4C).

As PAR-1 was found to be expressed in reactive astrocytes in the injured cord, we next examined the severity of glial scar formation at 42 days after injury by evaluating the complexity and extent of the astrocytic scarring using a semi-quantitative scale. Reactive astrocytes, demonstrated by GFAP immunostaining, aggregated to form a glial scar along the lesion border in both groups of mice (Fig 4D and 4E). In each mouse, scores obtained from 3 sample sections of the spinal cord at intervals of 480 um across the lesion epicenter were totaled for comparison. The result showed that glial scarring was significantly reduced in injured PAR-1 null mice (47 ± 8) than in the wild-type mice (80 ± 9) (Fig 4F). Collectively, our results indicate that PAR-1 deficiency facilitates favorable wound healing after spinal cord injury, evidenced by improved motor function recovery, preservation of white matter, and decreased glial scarring in the lesion.

### While treatment with rAPC after spinal cord injury improves locomotor recovery in wild-type mice no change is seen in PAR-1 null mice

**Fig 5:**
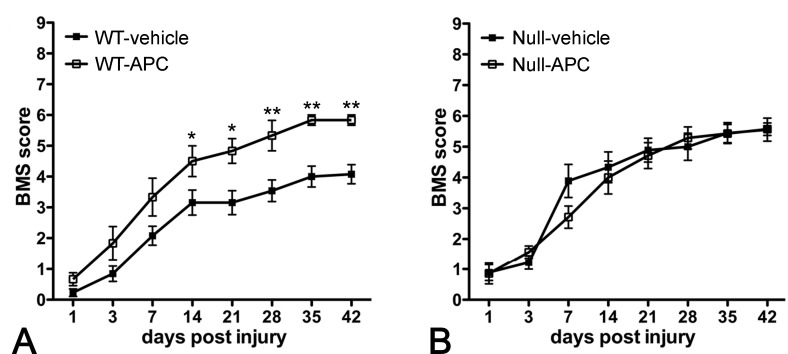
Improved locomotor recovery after the treatment with rAPC in spinal cord-injured wild-type mice. Administration of rAPC 20 minutes after the injury significantly improves locomotor performance of wild-type mice (A), starting from 14 days post injury, to an extent comparable to that of the injured PAR-1 null mice receiving no APC treatment. However, APC shows no additional benefit of functional improvements in injured PAR-1 null mice (B), suggesting that the effectiveness of APC is associated with PAR-1. (n = 10/genotype, means ± SEM, 2-way ANOVA,*p < 0.05, **p < 0.01).

We subsequently asked whether rAPC, given 20 minutes after injury, influences long-term locomotor recovery. We found that administration of rAPC to spinal cord-injured wild-type mice significantly improved locomotor recovery from 14 days after injury onwards as compared to vehicle-treated wild-type controls (Fig 5A). To determine if such benefit, afforded by rAPC treatment, is dependent upon the PAR-1 receptor, we examined locomotor recovery in spinal cord-injured PAR-1 null mice treated with or without rAPC. The results showed that the locomotor scores were comparable between these 2 groups, providing further evidence that PAR-1 is involved in APC-induced functional improvements (Fig 5B). Our findings suggest that APC facilitates locomotor recovery by counteracting PAR-1-mediated secondary injury after spinal cord injury.

## Discussion

The thrombin receptor PAR-1 is critical to a number of wound healing events after spinal cord injury. We demonstrate that spinal cord-injured PAR-1 null mice display reduced leukocyte infiltration, vascular barrier disruption, glial scar formation, and improved white matter sparing and locomotor recovery. We further find that injured wild-type mice treated with rAPC, presumably acting through the same PAR-1 receptor, improves neurological recovery to a similar degree to that of the injured PAR-1 null mice, suggesting that APC is acting through PAR-1 by the property of biased agonism. Together, these findings suggest that the PAR-1 receptor is an effective target for therapeutic intervention after spinal cord injury.

### Roles of PAR-1 in wound healing

Here we show that PAR-1 is expressed in neurons and blood vessels within the uninjured adult mouse spinal cord. Neuronal expression, particularly within alpha motoneurons and rat primary spinal afferent neurons, has been reported by others [42, 43]. PAR-1 has likewise been localized in blood vessels in the human brain [44]. Immunoreactivity of PAR-1 increased acutely within 24 hours after spinal cord injury, which is consistent with a rapid 12-fold increase in PAR-1 mRNA in the contused rat spinal cord [13]. This injury-induced PAR-1 immunoreactivity was primarily located in reactive astrocytes, suggesting PAR-1 has a functional role in response to neuropathological states.

Spinal cord injury is characterized by a breakdown in the blood-spinal cord barrier [37], resulting in an exodus of inflammatory cells and indiscriminate extravasation of molecules including plasma proteins to the cord parenchyma, which further damages the neuronal tissue [45, 46]. We have demonstrated reduced disruption of the blood-spinal cord barrier to the protein luciferase following spinal cord contusion in mice lacking the PAR-1 receptor. The dependency of barrier disruption on PAR-1 is mainly linked to thrombin. Research has shown that thrombin-triggered activation of PAR-1 results in disassembly of endothelial adhering junctions, altered endothelial morphology, and formation of inter-endothelial gaps [8, 47], giving rise to compromised barrier to circulating proteins and the evolution of vasogenic edema and neutrophil infiltration [18, 48].

Infiltration of neutrophils signals the early inflammatory response after spinal cord injury, which in turn is detrimental to the vasculature and spinal cord parenchyma, resulting in poor long-term locomotor recovery [28, 49]. This acute inflammation is mediated through PAR-1 via a variety of mechanisms. In addition to disruption of vascular architecture, PAR-1 increases neutrophil infiltration by promoting endothelial expression of surface P-selectin and subsequent leukocyte rolling in venules [50, 51]. PAR-1 also facilitates the release of inflammatory cytokines such as monocyte chemoattractant protein-1 (MCP-1) [52], TNF-alpha, interleukin-6 (IL-6), and IL-8 [53, 54].

After mechanical impact to the spinal cord, acute inflammation soon develops into prolonged secondary injuries, including macrophage infiltration, cell apoptosis, oxidative stress, demyelination, and axonal loss, which are more widespread than the original insult and finally lead to permanent functional disabilities [55, 56]. In injured PAR-1 null mice, reduced neutrophil infiltration early after the injury was closely associated with greater white matter sparing at the chronic stage of the injury, evidenced by the staining of myelinated axons with luxol fast blue. Therefore, significant attenuation of neutrophil infiltration and consequently better preserved white matter axons in PAR-1 null mice confirm the critical role of PAR-1 in de-stabilizing the blood-spinal cord barrier, which enhances early inflammation and ultimately contributes to white matter loss and locomotor deficits. Moreover, research has revealed that PAR-1 is a major suppressor of myelination during development and in adults. Deletion of PAR-1 enhances myelination as a result of elevated extracellular signal-regulated kinase (ERK)1/2 and AKT signaling and consequently more production of myelin basic protein, thicker myelin sheaths, and more myelinated axons in the spinal cord [57].

Whether reduced cell death in motor neurons contributes to better recovery of function in PAR-1 null mice is an intriguing question. Research has showed that the pro-apoptotic marker BCL2-interacting mediator of cell death is significantly lower at the lesion epicenter in PAR-1 null mice than in the wild-type mice 30 days after spinal cord injury [58]. However, more preserved neurons are observed in PAR-1 null mice only in the ventral horn at spinal segments rostral to the lesion epicenter, but not at the epicenter or caudal spinal segments [58]. This finding suggests that motor neurons in the ventral horn at the lesion epicenter are probably equally vulnerable to the contusion and consequent apoptosis in both PAR-1 null and wild-type mice. Therefore, the better recovery of motor function observed in injured PAR-1 null mice is more likely attributable to more spared white matter at the epicenter as demonstrated by our luxol fast blue staining, which has been showed to be the best single measurement for the severity of injury and predictive of motor recovery after contusive spinal cord injury in mice [59].

We found that PAR-1 was not expressed in astrocytes in the uninjured cord, but was up-regulated after a contusion injury primarily in reactive astrocytes with enhanced GFAP expression. After spinal cord injury, reactive astrocytes form a glial scar, which not only physically blocks regenerative axons but also biochemically arrests neurite outgrowth by its expression of growth inhibitory proteoglycans, leading to regenerative failure and defective locomotor function [60]. Here we show that PAR-1 null mice have reduced glial scar formation in the more chronically injured spinal cord, which is consistent with a reduced astrogliotic response to cortical stab wound [61] and spinal cord compression injury [58] in PAR-1 null mice, suggesting that PAR-1 is a determinant of glial scar formation.

In the injured spinal cord, the glial scar is formed by the migration and proliferation of reactive astrocytes around the lesion [62, 63]. Activation of PAR-1 not only mediates astrocytic secretion of anti-apoptotic cytokines *in vitro* [64] but also stimulates astrocyte proliferation and eventually astrogliosis after brain injury [61, 65]. The mechanisms underlying PAR-1-mediated astrocyte proliferation involve its coupling to multiple G-protein-linked signaling cascades, including Rho kinase, that induce sustained ERK activation [61, 66]. This sustained activation of ERK up-regulates astrocytic cyclin D1, a cell cycle modulator that controls cell proliferation [61]. Moreover, PAR-1 is known to mediate thrombin-induced expression of matrix metalloproteinase-9 [67], a zinc- and calcium-dependent endopeptidase that facilitates cell translocation by proteolytic remodeling of extracellular matrix molecules [68]. We have previously demonstrated that matrix metalloproteinase-9 not only enhances neutrophil infiltration and vascular breakdown [69] but also promotes astrocyte migration and glial scar formation after spinal cord injury [70]. Furthermore, astrocyte reactivity and migration in response to CNS insults is regulated by signal transducer and activator of transcription 3 (STAT3), which is activated by a number of cytokines including IL-6 [71, 72]. Recent research suggests that PAR-1 promotes astrocytic expression of IL-6, which in turn triggers STAT3 signaling cascade, causing enhanced migration of reactive astrocytes and astrogliosis after spinal cord injury [58]. Although the glial scar ultimately becomes an inhibitory barrier that blocks axonal regeneration at the chronic stage of the injury, its formation is considered beneficial at the acute stage by confining inflammation to the lesion epicenter and thus preventing the uninjured neuronal tissue from secondary damage [71, 73, 74]. The expression of PAR-1 in astrocytes, therefore, is conceivable to mediate both glial scar formation and inflammatory response during wound healing after spinal cord injury.

### PAR-1, APC, and motor function recovery

PAR-1 null mice showed significant locomotor improvement after spinal cord injury. This result highlights the importance of thrombin-PAR-1 as an upstream modulator of effects that adversely influence locomotor recovery. The mechanisms for improved locomotor recovery in spinal cord-injured PAR-1 null mice are likely significantly decreased inflammation, blood-spinal cord barrier breakdown, and/or glial scarring. As discussed above, the attenuation of early inflammation is possibly the most potent effect improving locomotor recovery because prolonged recruitment of neutrophils is associated with breakdown of the blood-brain barrier and a loss of the myelin sheaths from axons [75]. Similar improvements in both locomotion and myelin sparing have been demonstrated in spinal cord contusion models where early inflammation has been suppressed by anti CD 11d monoclonal antibodies [76]. Moreover, reduced glial scarring tempers the physical and biochemical barriers of astrogliosis to neuronal regeneration. Thus, absence of PAR-1 conceivably nullifies thrombin-induced neurological deficits, leading to substantially improved motor function. However, more research is needed to further explore other possible mechanisms underlying improved functional outcome when PAR-1 is eliminated.

For the first time in the setting of contusive spinal cord injury, we further demonstrated improvement in locomotor function after rAPC treatment in injured wild-type mice. APC and APC analogs have been proven effective in a number of disease states including spinal cord compression [28], spinal cord ischemia [77], traumatic brain injury [78], amyotrophic lateral sclerosis [79, 80], stroke [24, 81–83], murine sepsis [84], ischemic and reperfusion injury of heart [85], kidney, and liver [86], pulmonary, renal, and gastrointestinal inflammation [87], diabetes [88], and lethal body radiation [89]. However, until recently, the mechanism by which APC produced these beneficial effects across diverse pathologic states has not been entirely comprehended.

APC is effective against sepsis because it reduces inflammatory response by decreasing cytokine production, protecting endothelium against injury, and thus inhibiting neutrophil infiltration in mice [20, 90]. In endothelial cells and monocytes, APC reduces the expression of pro-inflammatory cytokines and apoptotic mediators by modulating nuclear factor-κB [91]. Moreover, the cytoprotective actions of APC require its ability to activate PAR-1 [30]. APC-PAR-1-mediated signaling is implicated in direct neuronal protection by inhibiting both the caspase-9 dependent and p53-mediated apoptotic pathways [26, 92]. In stroke models where the thrombolytic therapy of tissue plasminogen activator (tPA) is given, APC improves barrier function and blocks the complication of hemorrhage by inhibiting the activity of matrix metalloproteinase-9 [29, 93], which proteolytically degrades the vascular basement membrane and proteins associated with the blood-brain barrier [94].

We found that APC-treated wild-type mice showed better locomotor function than vehicle-treated wild-type mice, whereas APC-treated PAR-1 null mice exhibited comparable improvement to vehicle-treated PAR-1 null mice after spinal cord injury. These results demonstrated that APC acts on PAR-1, giving rise to beneficial outcome and, therefore, APC renders no additional improvement if PAR-1 is knocked out. Intriguingly, however, deletion of PAR-1 to block thrombin-mediated activation is also beneficial to functional recovery, suggesting a dual, ligand-dependent role of PAR-1 in wound healing after spinal cord injury.

The signaling mechanism by which APC uses the same PAR-1 receptor as thrombin to generate opposite effects has created tremendous interest. APC requires PAR-1 biased signaling to exhibit protective effects in endothelial cells, neurons, and microglia [24, 95]. PAR-1 signaling is initiated by different tethered N-terminal sequences, either the essential thrombin cleavage site Arg41 or the novel APC site Arg46 [96]. APC cleavage of PAR-1 signals B-arrestin-2, phosphatidylinositol 3-kinase/Akt, and Rac1, causing barrier protective and anti-inflammatory effects [30]. In endothelium, APC bound to endothelial protein C receptor (EPCR) uses PAR-1 to activate sphingosine 1-phosphate receptor 1 (S1P1). Cross-activation of S1P1 activates Rac1, leading to Rac1-dependent stabilization of the cytoskeleton [34]. This effect is opposite to the cleavage of PAR-1 by thrombin, which initiates GTPase RhoA and ERK1/2 signaling cascade, causing endothelial barrier disruption and proinflammatory effects [30, 32].

Therefore, biased agonism may explain what initially appears to be conflicting roles for PAR-1 observed in our study [30, 96, 97]. APC-PAR-1 signaling differs from thrombin-PAR-1 signaling in that thrombin cleaves and activates PAR-1 with at least 3 times greater efficiency [23]. Thus, PAR-1 signaling in the injured spinal cord is theoretically biased towards the abundant post-injury thrombin with consequent deleterious effects. When APC is administered following trauma, however, APC prevails and most likely becomes the dominant biased PAR-1 agonist, resulting in better protection of endothelial barrier and improved neurological outcome [98].

Further studies are needed to investigate the protective mechanism of APC-PAR-1 downstream signaling and to evaluate the optimal therapeutic window and pharmokinetics of APC treatment for spinal cord injury.

## Conclusion

In conclusion, we have demonstrated a detrimental role for thrombin-activated PAR-1 in wound healing and locomotor recovery after spinal cord injury. Administration of APC counteracts thrombin-PAR-1-induced adverse effects and provides protection. APC has been shown to exert beneficial effects in a spinal cord compression model in the rat [28] as well as in spinal cord ischemia in the rat and rabbit [77, 99]. Together with the current study, APC has now been independently validated in three species using different models of spinal cord injury. Understanding the mechanisms underlying the efficacy of APC provides opportunity for its further refinement with the goal of optimizing long-term neurological outcomes and translating this effort to human clinical trials.
